# Using mathematical models to optimise mosquito net distribution

**DOI:** 10.7554/eLife.112413

**Published:** 2026-08-07

**Authors:** Carlos A Prete

**Affiliations:** 1 School of Electrical and Computer Engineering, State University of Campinas Campinas Brazil

**Keywords:** malaria, mosquito net distribution, mathematical models, insecticide-treated nets, regional heterogeneity, *P. falciparum*

## Abstract

Tailoring malaria control interventions to regional transmission dynamics and behavioural characteristics can optimise them in resource-limited settings.

**Related research article** Glover AC, Koenker H, Niang EHA, Kolaczinski K, Churcher TS. 2026. Heterogeneity of use, access and retention of insecticide-treated nets: implications for subnational tailoring to maximise malaria control. *eLife* **14**:RP108745. doi: 10.7554/eLife.108745.

Malaria remains a major global health threat, causing over 600,000 deaths in 2024. Most deaths occur in sub-Saharan Africa, with children under five accounting for approximately 75% of these fatalities (World Health Organization, 2025b). The disease is transmitted through the bite of an *Anopheles* mosquito infected with a *Plasmodium* parasite. In sub-Saharan Africa, *Plasmodium falciparum* is the predominant malaria parasite and accounts for most malaria cases and deaths.

The distribution of insecticide-treated nets (ITNs) is one of the most effective malaria control interventions and is estimated to have averted 68% of all malaria cases between 2000 and 2015 (Bhatt et al., 2015). These nets are hung over beds to protect people from mosquito bites while they sleep and are treated with insecticides that kill mosquitoes that land on them.

ITNs are distributed by national malaria programmes through mass campaigns and continuous distribution channels, including antenatal care clinics, immunisation programmes, schools, and the private sector (Koenker et al., 2022; World Health Organization, 2025a). Although ITN distribution is a cost-effective intervention for malaria prevention, its implementation poses several challenges.

ITNs have limited durability due to physical damage and declining insecticidal effectiveness and therefore need to be replaced periodically. Mass distribution campaigns have traditionally been conducted on a triennial cycle, allowing periodic replacement of ITNs. However, individuals may stop using their ITNs before three years, potentially creating gaps in protection that could be prevented with more frequent campaigns. Moreover, access to ITNs does not necessarily translate into use: while 68% of households in malaria-endemic countries in Sub-Saharan Africa owned at least one ITN by 2024, only 47% of people reported sleeping under one (World Health Organization, 2025b). Finally, decades of use of nets treated with pyrethroids (a type of insecticide) have led to the emergence of pyrethroid-resistant mosquitoes. In response, the World Health Organization (WHO) recommends distributing ITNs containing two different insecticides (dual-active-ingredient ITNs), such as pyrethroid-chlorfenapyr ITNs, which are more expensive than pyrethroid-only ITNs (World Health Organization, 2025a).

National malaria programs are facing budget constraints and growing pressure to tailor ITN distribution to local conditions. Optimising interventions within limited budgets is therefore key to reducing transmission intensity, as the optimal intervention mix may vary with local transmission intensity, ITN use, and retention time. Now, in eLife, Andrew Glover and colleagues at Imperial College London, REACH Malaria, Université Cheikh Anta Diop and The Global Fund to Fight AIDS, Tuberculosis and Malaria, report on a mathematical model that uses Demographic and Health Survey (DHS) data to measure the impact of net use (Glover et al., 2026).

The researchers analysed data from 2005–2024 to estimate ITN use, access and use among people with access to a net (“use given access”), as well as the mean duration of use across regions in six African countries: Burkina Faso, Ghana, Malawi, Mali, Mozambique and Senegal. Comparing surveys conducted at different points in the distribution cycle may create misleading trends, since a survey conducted soon after a mass campaign is more likely to record higher ITN use. The researchers used a hierarchical Bayesian model to account for these differences in the timing of surveys and mosquito net distribution campaigns. The resulting estimates were fed to an individual-based malaria transmission model to estimate the number of cases averted under different interventions.

Glover et al. found that, on average, mosquito nets are kept for 27 months but used for only 21 months (though these estimates vary substantially across regions) – well short of the 3-year interval between campaigns. Reducing this interval to two years would increase average ITN use from 45.4% to 53.9%. However, for 75% of regions, the model also predicts that distributing pyrethroid-chlorfenapyr ITNs with triennial mass campaigns averts more malaria cases than distributing pyrethroid-only ITNs with biennial mass campaigns.

The model highlights substantial regional heterogeneity in ITN use and retention, suggesting that the most beneficial intervention may differ across regions. In locations with high use given access and longer retention time, ITN distribution is more likely to be effective, while regions with high use given access but low retention time would benefit from shorter intervals between mass campaigns. By contrast, in regions where access is high, but use remains low, social and behaviour change campaigns to increase ITN use might be more effective. Notably, although the WHO previously recommended that at least 80% of people in at-risk populations use ITNs, none of the regions studied achieved this average level of ITN use (Roll Back Malaria Partnership, 2008; Smith et al., 2009).

The model estimates that discontinuing mass campaigns and relying exclusively on continuous distribution channels could lead to substantial malaria resurgence, even if more effective dual-ingredient ITNs are distributed through continuous channels. Furthermore, the model indicates that regions with higher ITN use given access are disproportionately affected by the cessation of ITN distribution, and that use given access is a much stronger predictor of this impact than ITN use alone.

The work of Glover et al. demonstrates how tailoring malaria control interventions to regional transmission dynamics and behavioural characteristics can optimise interventions in settings with limited resources and highlights the importance of mathematical models for more informed decision-making.
